# Historical Changes in Weight Classes and the Influence of NAFLD Prevalence: A Population Analysis of 34,486 Individuals

**DOI:** 10.3390/ijerph19169935

**Published:** 2022-08-11

**Authors:** Benjamin Kai Yi Nah, Cheng Han Ng, Kai En Chan, Caitlyn Tan, Manik Aggarwal, Rebecca Wenling Zeng, Jieling Xiao, Yip Han Chin, Eunice X. X. Tan, Yi Ping Ren, Douglas Chee, Jonathan Neo, Nicholas W. S. Chew, Michael Tseng, Mohammad Shadab Siddiqui, Arun J. Sanyal, Yock Young Dan, Mark Muthiah

**Affiliations:** 1 Division of Gastroenterology and Hepatology, Department of Medicine, National University Hospital, Singapore 119074, Singapore; 2 MBBS Programme, Yong Loo Lin School of Medicine, National University of Singapore, Singapore 119077, Singapore; 3 Department of Gastroenterology and Hepatology, Mayo Clinic, Rochester, MN 55902, USA; 4 National University Centre for Organ Transplantation, National University Health System, Singapore 119228, Singapore; 5 Department of Internal Medicine, National University Hospital, Singapore 119074, Singapore; 6 Department of Cardiology, National University Heart Centre, National University Hospital, Singapore 119074, Singapore; 7 Division of Gastroenterology, Hepatology and Nutrition, Department of Internal Medicine, Virginia Commonwealth University, Richmond, VA 23284, USA

**Keywords:** non-alcoholic fatty liver disease, weight classes, body mass index

## Abstract

**Background:** Non-alcoholic fatty liver disease (NAFLD) is the most common chronic liver disease globally in tandem with the growing obesity epidemic. However, there is a lack of data on the relationship between historical weight changes 10 years ago and at present on NAFLD prevalence at the population level. Therefore, we sought to evaluate the relationship between weight classes and the prevalence of NAFLD. **Methods:** Data were used from the United States National Health and Nutrition Examination Survey (NHANES) from 1999 to 2018. Univariate and multivariate general linear model analyses were used to obtain risk ratio (RR) estimations of NAFLD events. **Results**: In total, 34,486 individuals were analysed, with those who were lean at both time points as the control group. Overweight (RR: 14.73, 95%CI: 11.94 to 18.18, *p* < 0.01) or obese (RR: 31.51, 95%CI: 25.30 to 39.25, *p* < 0.01) individuals at both timepoints were more likely to develop NAFLD. Residual risk exists where previously obese individuals became overweight (RR: 14.72, 95%CI: 12.36 to 17.52, *p* < 0.01) or lean (RR: 2.46, 95%CI: 1.40 to 4.31, *p* = 0.02), and previously overweight individuals who became lean (RR 2.24, 95%CI 1.42 to 3.54, *p* = 0.01) had persistent elevated risk of developing NAFLD despite weight regression. Sensitivity analysis identified that a higher proportion of individuals with regression in weight class were diabetics and Mexican Americans, while fewer African Americans saw weight-class regression. **Conclusions**: Residual risk exists in patients who lost weight despite the smaller magnitude of effect, and targeted weight reductions should still be used to mitigate the risk of NAFLD at the population level.

## 1. Introduction

Non-alcoholic fatty liver disease (NAFLD) is the most prevalent chronic liver disease, with recent estimates suggesting that upwards of 25 to 33 percent [1,2,3] of the global population is affected by the disease. NAFLD is defined by the presence of significant (>5%) hepatic steatosis in the absence of significant alcohol intake and other secondary causes of hepatic steatosis. Histologically, NAFLD can be subdivided by severity into non-alcoholic fatty liver (NAFL) or non-alcoholic steatohepatitis (NASH), with the latter characterised by the presence of lobar inflammation and ballooning, with or without liver fibrosis [4]. NAFLD is closely associated with features of metabolic syndrome, and these conditions have been shown to have a multiplicative effect on NAFLD severity. Obesity is a major risk factor for NAFLD, where an increase in visceral fat promotes insulin resistance [5,6]. Insulin resistance leads to an increased delivery of free fatty acids to the liver, which in turn drives hepatic steatosis. Additionally, a shift towards a pro-inflammatory state has been associated with chronic liver inflammation and fibrosis through the upregulation of proinflammatory cytokines, such as leptin, and the inhibition of the anti-inflammatory cytokine adiponectin [7,8]. Despite the significant burden of NAFLD, there is currently no established treatment for NAFLD, and moderation of the disease has largely relied on dietary changes and increased physical activity. The contribution of obesity and weight in NAFLD is significant, and weight loss has demonstrated significant improvements in liver histology in NASH patients.

Although current literature has demonstrated a strong association between weight gain later in adult life and a higher risk of cardiometabolic diseases, obesity-related malignancies, and death [9], the relationship between weight changes and the risk of incident NAFLD is less understood. In the Multicentre Coronary Artery Risk Development cohort (CARDIA Cohort) [10], participants with weight gain were found to have significantly greater odds of developing NAFLD compared to individuals with unchanged weight. Larger studies in exclusively Asian cohorts have also shown similar results, where over a median follow-up time of 5.2 years of individuals with body mass index (BMI) > 23 kg/m^2^, Cho et al. [11] found a higher risk of developing NAFLD in individuals that experienced weight gain compared to individuals with limited weight change. With the increasing obesity epidemic and growing NAFLD globally, especially in the United States (US), there is a need for representative, population-level data to assess the impact of weight pattern changes on risk of NAFLD development. Thus, the current study seeks to examine the risk of NAFLD based on changes in weight classes over a 10-year period.

## 2. Methods

The NHANES study examines aggregated health-related data from a cluster sample national survey involving general and noninstitutionalised individuals in the United States between 1999 and 2018. In view of the weight collected from participants before and after 10 years, only individuals aged 30 to 80 were included in this study to prevent inclusion of the paediatric population. The study involved participants undergoing medical examinations, laboratory assessments, and comprehensive interviews that included recall questions to establish a retrospective, longitudinal cohort based on the cross-sectional data. Ethics approval by the Institutional Review Board was exempted due to the anonymous nature of the data made publicly available by the National Centre for Health Statistics (NCHS). Baseline characteristics such as, but not limited to, age, gender, ethnicity, body mass index (BMI), waist circumference, low-density lipoprotein (LDL) cholesterol, high-density lipoprotein (HDL) cholesterol, total cholesterol, triglyceride, total bilirubin, fasting blood glucose, glycohemoglobin, aspartate aminotransferase (AST), alanine aminotransferase (ALT), and past medical history (diabetes mellitus and hypertension) were collected. Individuals were subdivided into nine BMI-change patterns according to the individual’s weight trend, with reference to anthropometrics that were recorded at the time of the study and based on participants’ recollection of their weight 10 years prior. Information on the outcomes of NAFLD across the various BMI-change patterns was also collected.

The Fatty Liver Index (FLI) was used to determine NAFLD status using BMI, waist circumference (WC), triglycerides (TG), and gamma-glutamyl transferase (GGT). The FLI equation is as follows: FLI = (e^0.953×oge(TG)+0.139×BMI+0.718×loge(GGT)+0.053×WC−15.745^)/(1 + e^0.953×loge(TG)+0.139×BMI+0.718×loge(GGT)+0.053×WC−15.745^) × 100 [12]. The FLI classifies subjects with a score of >60 or US-FLI (US FLI) ≥30 without significant alcohol consumption as having NAFLD, as per AASLD Practice Guidance [13]. Overweight individuals were defined as BMI ≥ 25.0 kg/m^2^ for Caucasians and BMI ≥ 23.0 kg/m^2^ for Asians [14], and obese individuals were defined as BMI ≥ 30.0 kg/m^2^ for Caucasians and BMI ≥ 27.5 kg/m^2^ for Asians [14]. The remaining individuals were defined as lean. Weight regressors were defined as individuals who decreased their BMI category (e.g., obese to lean or obese to overweight) over the 10-year period. Weight progressors were defined as individuals who had an increase in their BMI category (e.g., lean to overweight or lean to obese) over 10 years. Individuals with maintenance of weight class were defined as individuals who remained in the same BMI category at both time points (e.g., lean to lean, obese to obese). Diabetes was defined as glycohemoglobin ≥ 6.5%, fasting plasma glucose ≥ 7 mmol/L [15], self-reported diabetes, or the use of anti-diabetic medications. Hypertension was defined as a systolic or diastolic blood pressure ≥ 140/90 mmHg [16] or use of antihypertensives.

All statistical analysis was performed using STATA (17.0), with individuals identified as lean at both timepoints as the reference group. Continuous variables were examined with the Wilcoxon ranked sum test and the Kruskal–Wallis analysis of variance while binary variables were examined with the chi-square test and Fisher’s exact test where appropriate. Univariate and multivariate general linear model analysis with a log link, Gaussian family estimation, and robust variance estimation were used to obtain relative risk estimations of NAFLD events amongst the various BMI-change patterns. Multivariate analysis in general linear model analysis was constructed with important traditional confounders that include age, gender, ethnicity, diabetes, hypertension, and a cluster variable on the year of study. Subgroup analysis of individuals with NAFLD was conducted with sensitivity analysis to compare between weight regressors, weight progressors, and individuals with maintenance of weight class.

## 3. Results

### 3.1. Baseline Characteristics of Included Population

In total, 34,486 individuals were included in the analysis. Based on self-reported weight 10 years prior to the study, 12,239 individuals were identified as lean, 12,800 were overweight, and 9447 individuals were categorised as obese. At the time of study, 8555 individuals were identified as lean, 12,345 were overweight, and 13,586 individuals were categorised as obese. A summary of the clinical characteristics of lean, overweight, and obese individuals 10 years prior and at the time of study can be found in Supplementary Table S1 and Table 1, respectively. At both timepoints of the study, there were similar differences in baseline characteristics, although there were substantially more overweight and obese individuals 10 years later. Unsurprisingly, the obese population had the worst lipid profiles, highest fasting glucose, and highest prevalence of diabetes and hypertension out of the three weight classes.

### 3.2. Weight Changes and Risk of NAFLD

**Figure 1:**
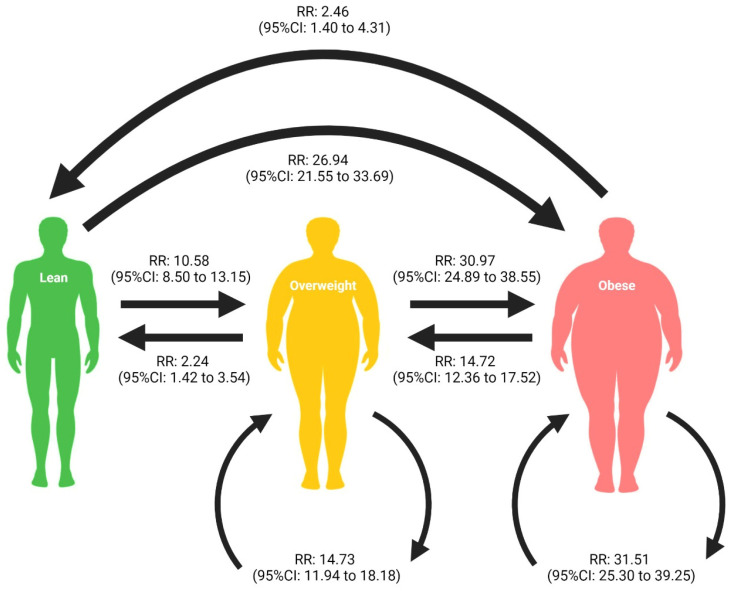
Relative Risk of NAFLD Development Across Different Weight-Change Patterns.

A generalised linear model was used to estimate the risk of developing NAFLD across the various weight-change patterns; a summary of the relative risk of NAFLD is presented in Figure 1. Progression of weight class significantly increased the risk of NAFLD. Compared to individuals identified as lean at both timepoints, individuals who were lean 10 years ago but became overweight (RR: 10.58, 95%CI: 8.50 to 13.15, *p* < 0.01) or obese (RR 26.94, 95%CI: 21.55 to 33.69, *p* < 0.01) at the time of study had a significantly higher risk of developing NAFLD. Individuals who were previously overweight 10 years ago but became obese had an increased risk of NAFLD (RR: 30.97, 95%CI: 24.89 to 38.55, *p* < 0.01). However, individuals who maintained their weight classes were also at a significantly increased risk of NAFLD, with the exception of those who were identified as lean at both timepoints. The individuals that identified as overweight (RR: 14.73, 95%CI: 11.94 to 18.18, *p* < 0.01) or obese (RR: 31.51, 95%CI: 25.30 to 39.25, *p* < 0.01) 10 years ago and at the time of study were statistically more likely to develop NAFLD compared to individuals identified as lean at both timepoints. While weight reduction is known to reduce the risk of NAFLD, regression in weight classes did not completely eliminate the risk of NAFLD. Lean individuals who were previously overweight (RR: 2.24, 95%CI 1.42 to 3.54, *p* = 0.01) or obese (RR: 2.46, 95%CI: 1.40 to 4.31, *p* = 0.02) 10 years ago had a significantly elevated risk of developing NAFLD despite regression in their weight classes.

### 3.3. Factors Associated with Weight Regression

A sensitivity analysis was performed amongst 9399 individuals with NAFLD to compare characteristics amongst weight regressors to individuals who maintained or progressed in weight class (Table 2). Only 5.08% of NAFLD patients were weight regressors, while 56.46% maintained weight class and 38.46% progressed in weight class. Older adults were found to be more likely to regress in weight in comparison, and, interestingly, diabetes mellitus was more prevalent in weight regressors compared to individuals with maintenance of weight class and weight progressors (40.52%, 95%CI: 36.12 to 45.08 vs. 27.94%, 95%CI: 27.00 to 28.89, *p* < 0.01). Additionally, there was a higher proportion of Mexican Americans amongst weight regressors compared to individuals with maintenance of weight class and weight progressors (29.56%, 95%CI: 25.63 to 33.81 vs. 17.59%, 95%CI: 16.81 to 18.39, *p* < 0.01). Conversely, there was a lower proportion of African Americans amongst in weight regressors compared to individuals with maintenance of weight class and weight progressors (13.00%, 95%CI: 10.27 to 16.32 vs. 21.62%, 95%CI: 20.78 to 22.49, *p* < 0.01). A higher annual household income is associated with maintenance of weight class and weight progressors. Amongst individuals with maintenance of weight class and weight progressors, 21.43% (95%CI: 20.51 to 22.38, *p* < 0.01) and 23.40% (95%CI: 22.45 to 24.37, *p* < 0.01) of individuals had an annual household income of ≥USD 75,000 and USD 45,000 to USD 74,999, respectively, compared to 16.67% (95%CI: 13.36 to 20.60, *p* < 0.01) and 18.63% (95%CI: 15.14 to 22.70, *p* < 0.01) of individuals who were weight regressors.

## 4. Discussion

While obesity is a major risk factor for NAFLD development [4,17], there has yet to be representative population-wide evidence on the effects of weight change on the prevalence of NAFLD. Such population results are vital to better our understanding of a crucial risk factor for NAFLD development, thereby facilitating the institution of national-level policies and measures to combat the major public health risk. While previous studies by VanWagner et al. [10] demonstrated the association of weight gain with an increased risk of NAFLD, more than half of the study population were only lean individuals, and there was a lack of data on the association of weight change and risk of NAFLD in overweight or obese individuals. Furthermore, there remains a paucity of studies on the effects of weight class regression on NAFLD development. Thus, our study adds to the current literature by presenting the association of weight progression, maintenance, and regression on the risk of NAFLD development in individuals of different weight classes at the population level. With the use of the population-wide NHANES cohort, these results are generalizable to the US population at large and can be used to inform future policy decisions.

In our present analysis, individuals who were overweight or obese were at higher risk of developing NAFLD compared to lean individuals. However, overweight and obese individuals who experienced no change in their BMI category over the 10-year follow-up period were associated with a significant increased risk of NAFLD relative to lean NAFLD whose BMI category did not change. This is unsurprising, as obesity is an independent predictor for increased visceral adiposity [18], resulting in insulin resistance, increased lipolysis, and persistently elevated serum levels of free fatty acids. Subsequently, a multitude of mechanisms including the dysregulation of adipokines, lipotoxicity, release of pro-inflammatory cytokines, and oxidative stress may ultimately contribute to a state of intrahepatic fat accumulation and chronic inflammation characteristic of NAFLD [19,20]. While the merits of weight loss in histological improvement of NASH and NAFLD regression is well-demonstrated in previous studies [21,22], the influence of weight loss on the risk of developing incident NAFLD is less well-understood. Our study shows that patients previously classified as overweight (RR: 2.24, 95%CI: 1.42 to 3.54, *p* = 0.01) or obese (RR: 2.46, 95%CI: 1.40 to 4.31, *p* = 0.02) individuals who subsequently became lean still had an elevated risk of developing incident NAFLD compared to individuals who were lean at both timepoints. This is likely the result of residual risks associated with a previous history of obesity and may be due to the preferential reduction in subcutaneous adipose tissue as opposed to visceral adipose tissue during weight loss. Visceral adipose tissue is associated with increased insulin resistance [23] and is a key driver for development of NAFLD. However, the relative magnitude of the risk of developing NAFLD after losing weight compared to individuals who maintained their weight classes is still significantly smaller and the importance of weight loss should not be understated.

Concerningly, however, only 5.35% of individuals with NAFLD were classified as weight regressors, and ethnicity, annual household income, and presence of diabetes mellitus were found to be potential factors that influence weight regression. Weight perception in NAFLD has been a major limitation, and findings from the current study show that individuals of Mexican American ethnicity are associated with regression in weight class whilst individuals of African American ethnicity may find it more difficult to lose weight. The finding may be explained by different perspectives amongst racial and ethnic groups on limited work–life flexibility, convenience of evidence-based diabetes prevention classes, and availability of disposable income to purchase supplementary resources. Additionally, a significantly higher proportion of individuals with diabetes had weight class regression, but the finding could be confounded by the use of certain classes of anti-diabetic medications, such as biguanides, glucagon-like peptide-1, and sodium-glucose cotransporter inhibitors [24], or may be influenced by the presence of dietitians accessible to diabetics.

## 5. Limitations

The present study evaluates the relationship between weight trends over 10 years and its impact on the prevalence of NAFLD through a population analysis of 34,486 individuals. However, there are several limitations in this study. Self-reported data on patients’ weight 10 years prior to the time of survey may be subjected to recall bias. However, several validation studies have suggested that self-reported weight was strongly correlated with anthropometric measures and could be used in life course epidemiology studies [25,26]. Additionally, the time of onset of NAFLD was not accounted for in the present analysis, which may confound the effect of weight change on the development of NAFLD. Furthermore, the NHANES dataset is a cross-sectional examination of NAFLD patients, thus limiting the potential for temporal causality inference or longitudinal follow up of NAFLD patients’ weight changes. As not all NAFLD patients in this dataset were suitable candidates for the Dual-energy X-ray Absorptiometry (DXA) scan, measurements such as lean body mass and muscle mass were not included in the present study and detailed changes in body composition could not be analysed. Lastly, insufficient longitudinal data regarding the patients’ medication profiles may be a potential confounder of the study results.

## 6. Conclusions

This study reports on the relationship between weight classes and the prevalence of NAFLD as well as factors associated with weight class regression using multi-ethnic US population data. Weight remains to be a major modulator of NAFLD prevalence. While regression in weight classes does not completely eliminate the risk of NAFLD, strong emphasis on health-promotion activities such as weight loss programmes should continue to mitigate the risk of NAFLD development and improve overall cardiovascular health. Additionally, public health stakeholders should be mindful of the complex influence of socioeconomic and environmental factors amongst ethnic classes on the efficacy of health-promotion activities to ensure easy and equal accessibility for all.

## Figures and Tables

**Table 1:** Clinical Characteristics of Lean, Overweight, and Obese Population at Time of Survey.

	Lean	Overweight	Obese	*p*-Value
Sample Size	8555	12,345	13,586	
Age (years)	56.00 (IQR: 45.00 to 69.00)	58.00 (IQR: 46.00 to 69.00)	56.00 (IQR: 46.00 to 66.00)	<0.01 *
Gender (male)	45.86 (95%CI: 44.80 to 46.91)	56.67 (95%CI: 55.79 to 57.54)	44.54 (95%CI: 43.70 to 45.38)	<0.01 *
Platelet (1000 cells/uL)	240.00 (IQR: 203.00 to 286.00)	239.00 (IQR: 201.00 to 281.00)	247.00 (IQR: 207.00 to 294.00)	<0.01 *
Glycohemoglobin (%)	5.40 (IQR: 5.20 to 5.70)	5.60 (IQR: 5.30 to 5.90)	5.70 (IQR: 5.40 to 6.30)	< 0.01 *
Fasting Glucose (mmol/L)	5.38 (IQR: 5.05 to 5.86)	5.66 (IQR: 5.27 to 6.22)	5.94 (IQR: 5.42 to 6.83)	<0.01 *
Total Bilirubin (umol/L)	10.30 (IQR: 8.55 to 13.68)	10.30 (IQR: 8.55 to 13.68)	10.26 (IQR: 6.84 to 13.68)	<0.01 *
AST (IU/L)	23.00 (IQR: 19.00 to 27.00)	23.00 (IQR: 20.00 to 28.00)	23.00 (IQR: 19.00 to 28.00)	<0.01 *
ALT (IU/L)	19.00 (IQR: 15.00 to 24.00)	21.00 (IQR: 17.00 to 28.00)	22.00 (IQR: 17.00 to 31.00)	<0.01 *
GGT (IU/L)	18.00 (IQR: 13.00 to 27.00)	22.00 (IQR: 15.00 to 33.00)	24.00 (IQR: 17.00 to 37.00)	<0.01 *
LDL (mg/dL)	113.00 (IQR: 92.00 to 138.00)	119.00 (IQR: 95.00 to 143.00)	115.00 (IQR: 92.00 to 139.00)	<0.01 *
HDL (mg/dL)	60.00 (IQR: 49.00 to 73.00)	50.00 (IQR: 42.00 to 62.00)	47.00 (IQR: 40.00 to 57.00)	<0.01 *
Total Cholesterol (mg/dL)	197.00 (IQR: 172.00 to 224.00)	200.00 (IQR: 174.00 to 229.00)	195.00 (IQR: 169.00 to 224.00)	<0.01 *
Triglycerides (mg/dL)	98.00 (IQR: 69.00 to 145.00)	131.00 (IQR: 89.00 to 198.00)	145.00 (IQR: 100.00 to 214.00)	<0.01 *
Waist Circumference (cm)	84.30 (IQR: 78.70 to 89.90)	97.30 (IQR: 92.30 to 102.40)	112.20 (IQR: 105.40 to 120.70)	<0.01 *
Body Mass Index (kg/m^2^)	22.60 (IQR: 21.03 to 23.90)	27.30 (IQR: 26.10 to 28.60)	33.94 (IQR: 31.60 to 37.80)	<0.01 *
Weight (kg)	61.70 (IQR: 55.30 to 68.40)	76.50 (IQR: 69.20 to 84.00)	95.80 (IQR: 85.30 to 108.40)	<0.01 *
Diabetes	11.04 (95%CI: 10.37 to 11.74)	18.11 (95%CI: 17.43 to 18.82)	31.39 (95%CI: 30.59 to 32.21)	<0.01 *
Hypertension	51.87 (95%CI: 50.75 to 52.99)	61.19 (95%CI: 60.29 to 62.09)	72.53 (95%CI: 71.74 to 73.30)	<0.01 *
Ethnicity				<0.01 *
Mexican American	11.23 (95%CI: 10.58 to 11.92)	18.06 (95%CI: 17.39 to 18.74)	17.31 (95%CI: 16.68 to 17.96)	
Hispanic	6.95 (95%CI: 6.43 to 7.51)	9.12 (95%CI: 8.63 to 9.64)	8.27 (95%CI: 7.82 to 8.75)	
Caucasian	51.23 (95%CI: 50.17 to 52.29)	44.73 (95%CI: 43.86 to 45.61)	42.32 (95%CI: 41.49 to 43.15)	
African American	18.38 (95%CI: 17.57 to 19.21)	18.38 (95%CI: 17.71 to 19.07)	25.61 (95%CI: 24.88 to 26.35)	
Other Race	12.20 (95%CI: 11.53 to 12.91)	9.71 (95%CI: 9.20 to 10.25)	6.49 (95%CI: 6.09 to 6.92)	
Annual Household Income				<0.01 *
<USD 10,000	8.10 (95%CI: 7.48 to 8.76)	6.58 (95%CI: 6.11 to 7.07)	7.45 (95%CI: 6.99 to 7.95)	
USD 10,000–24,999	26.38 (95%CI: 25.36 to 27.42)	25.11 (95%CI: 24.28 to 25.96)	26.12 (95%CI: 25.32 to 26.93)	
USD 25,000–44,999	23.69 (95%CI: 22.71 to 24.70)	25.05 (95%CI: 24.22 to 25.90)	25.02 (95%CI: 24.24 to 25.82)	
USD 45,000–74,999	20.35 (95%CI: 19.42 to 21.31)	22.52 (95%CI: 21.72 to 23.34)	21.98 (95%CI: 21.23 to 22.75)	
≥USD 75,000	21.48 (95%CI: 20.53 to 22.46)	20.75 (95%CI: 19.97 to 21.54)	19.42 (95%CI: 18.71 to 20.16)	

**Legend:** IQR, Interquartile Range; 95%CI, 95% Confidence Interval; AST, Aspartate Aminotransferase; ALT, Alanine Aminotransferase; GGT, Gamma-Glutamyl Transferase; LDL, Low-Density Lipoprotein; HDL, High-Density Lipoprotein. * bolded *p*-value ≤ 0.05 denotes statistical significance.

**Table 2:** Clinical Characteristics of Weight Regressors versus Individuals with Maintenance of Weight Class and Weight Progressors in a Population with NAFLD.

	Weight Regressors	Non-Weight Regressors	*p*-Value
Sample Size	477	8922	
Age (years)	64.00 (IQR: 55.00 to 72.00)	58.00 (IQR: 47.00 to 67.00)	<0.01 *
Gender (male)	61.01 (95%CI: 56.55 to 65.29)	49.14 (95%CI: 48.10 to 50.17)	<0.01 *
Platelet (1000 cells/uL)	229.50 (IQR: 187.00 to 278.00)	247.00 (IQR: 208.00 to 292.00)	<0.01 *
Glycohemoglobin (%)	5.80 (IQR: 5.40 to 6.60)	5.70 (IQR: 5.40 to 6.20)	<0.01 *
Fasting Glucose (mmol/L)	6.23 (IQR: 5.55 to 7.77)	5.94 (IQR: 5.45 to 6.77)	<0.01 *
Total Bilirubin (umol/L)	10.26 (IQR: 8.55 to 13.68)	10.26 (IQR: 8.55 to 13.68)	**0.02**
AST (IU/L)	24.00 (IQR: 20.00 to 29.00)	23.00 (IQR: 20.00 to 28.00)	0.20
ALT (IU/L)	22.00 (IQR: 17.00 to 28.00)	23.00 (IQR: 18.00 to 31.00)	<0.01 *
GGT (IU/L)	29.00 (IQR: 20.00 to 43.00)	25.00 (IQR: 18.00 to 38.00)	<0.01 *
LDL (mg/dL)	114.00 (IQR: 88.00 to 142.00)	118.00 (IQR: 95.00 to 143.00)	0.06
HDL (mg/dL)	46.00 (IQR: 39.00 to 55.00)	46.00 (IQR: 39.00 to 55.00)	<0.01 *
Total Cholesterol (mg/dL)	196.00 (IQR: 172.00 to 230.00)	200.00 (IQR: 174.00 to 229.00)	0.40
Triglycerides (mg/dL)	176.00 (IQR: 129.00 to 271.00)	160.00 (IQR: 112.00 to 232.00)	<0.01 *
Waist Circumference (cm)	101.70 (IQR: 96.90 to 106.90)	109.50 (IQR: 103.00 to 118.00)	<0.01 *
Body Mass Index (kg/m^2^)	28.51 (IQR: 26.97 to 29.93)	32.64 (IQR: 29.86 to 36.50)	<0.01 *
Weight (kg)	78.60 (IQR: 70.00 to 87.10)	92.10 (IQR: 81.80 to 104.60)	<0.01 *
Diabetes	40.52 (95%CI: 36.12 to 45.08)	27.94 (95%CI: 27.00 to 28.89)	<0.01 *
Hypertension	76.77 (95%CI: 72.65 to 80.43)	71.71 (95%CI: 70.73 to 72.67)	**0.02**
Ethnicity			<0.01 *
Mexican American	29.56 (95%CI: 25.63 to 33.81)	17.59 (95%CI: 16.81 to 18.39)	
Hispanic	5.87 (95%CI: 4.08 to 8.37)	8.36 (95%CI: 7.80 to 8.95)	
Caucasian	46.54 (95%CI: 42.10 to 51.03)	46.27 (95%CI: 45.23 to 47.30)	
African American	13.00 (95%CI: 10.27 to 16.32)	21.62 (95%CI: 20.78 to 22.49)	
Other Race	5.03 (95%CI: 3.39 to 7.40)	6.16 (95%CI: 5.68 to 6.68)	
Annual Household Income			<0.01 *
<USD 10,000	8.82 (95%CI: 6.43 to 11.99)	5.89 (95%CI: 5.37 to 6.45)	
USD 10,000–24,999	26.96 (95%CI: 22.88 to 31.48)	24.02 (95%CI: 23.06 to 25.00)	
USD 25,000–44,999	28.92 (95%CI: 24.73 to 33.51)	25.27 (95%CI: 24.30 to 26.28)	
USD 45,000–74,999	18.63 (95%CI: 15.14 to 22.70)	23.40 (95%CI: 22.45 to 24.37)	
≥USD 75,000	16.67 (95%CI: 13.36 to 20.60)	21.43 (95%CI: 20.51 to 22.38)	

**Legend:** IQR, Interquartile Range; 95%CI, 95% Confidence Interval; AST, Aspartate Aminotransferase; ALT, Alanine Aminotransferase; GGT, Gamma-Glutamyl Transferase; LDL, Low-Density Lipoprotein; HDL, High-Density Lipoprotein, * bolded *p*-value ≤ 0.05 denotes statistical significance.

## Data Availability

Data were retrieved from the National Health and Nutrition Examination Survey Registry.

## Supplementary Materials

The following supporting information can be downloaded at: https://www.mdpi.com/article/10.3390/ijerph19169935/s1, Table S1: Clinical Characteristics of Lean, Overweight and Obese Population 10 Years Prior.
