# Supplementary material for: Historical Changes in Weight Classes and the Influence of NAFLD Prevalence: A Population Analysis of 34,486 Individuals
Source: Int J Environ Res Public Health. 2022 Aug 11;19(16):9935. doi: 10.3390/ijerph19169935 (PMC9408006; doi:10.3390/ijerph19169935)
Supplement: Supplementary file 1 [file ijerph-19-09935-s001.zip › Supplementary Table S1.pdf]

Supplementary Table S1. Clinical Characteristics of Lean, Overweight and Obese Population 10 Years Prior

	Lean	Overweight	Obese	P-value
Sample Size	12,239	12,800	9,447	
Age (years)	52.00 (IQR: 43.00 to 64.00)	59.00 (IQR: 47.00 to 69.00)	60.00 (IQR: 48.00 to 69.00)	**< 0.01***
Gender (male)	40.17 (95%CI: 39.31 to 41.05)	57.58 (95%CI: 56.72 to 58.43)	49.57 (95%CI: 48.56 to 50.58)	**< 0.01***
Platelet (1000 cells/uL)	248.00 (IQR: 209.00 to 292.00)	239.00 (IQR: 202.00 to 284.00)	239.00 (IQR: 200.00 to 285.00)	**< 0.01***
Glycohemoglobin (%)	5.50 (IQR: 5.20 to 5.70)	5.60 (IQR: 5.30 to 6.00)	5.80 (IQR: 5.50 to 6.50)	**< 0.01***
Fasting Glucose (mmol/L)	5.44 (IQR: 5.05 to 5.88)	5.77 (IQR: 5.33 to 6.42)	6.00 (IQR: 5.44 to 7.16)	**< 0.01***
Total Bilirubin (umol/L)	10.26 (IQR: 8.55 to 13.68)	10.26 (IQR: 8.55 to 13.68)	10.26 (IQR: 8.55 to 13.68)	**< 0.01***
AST (IU/L)	23.00 (IQR: 19.00 to 27.00)	23.00 (IQR: 20.00 to 28.00)	22.00 (IQR: 19.00 to 27.00)	**< 0.01***
ALT (IU/L)	20.00 (IQR: 16.00 to 26.00)	22.00 (IQR: 17.00 to 29.00)	21.00 (IQR: 16.00 to 29.00)	**< 0.01***
GGT (IU/L)	19.00 (IQR: 14.00 to 30.00)	23.00 (IQR: 16.00 to 34.00)	23.00 (IQR: 17.00 to 35.00)	**< 0.01***
LDL (mg/dL)	117.00 (IQR: 95.00 to 142.00)	118.00 (IQR: 95.00 to 142.00)	111.00 (IQR: 87.00 to 136.00)	**< 0.01***
HDL (mg/dL)	56.00 (IQR: 46.00 to 69.00)	49.00 (IQR: 41.00 to 60.00)	48.00 (IQR: 40.00 to 58.00)	**< 0.01***
Total Cholesterol (mg/dL)	201.00 (IQR: 176.00 to 228.00)	199.00 (IQR: 173.00 to 227.00)	190.00 (IQR: 163.00 to 219.00)	**< 0.01***
Triglycerides (mg/dL)	110.00 (IQR: 76.00 to 167.00)	135.00 (IQR: 92.00 to 204.00)	138.00 (IQR: 95.00 to 205.00)	**< 0.01***
Waist Circumference (cm)	89.40 (IQR: 81.90 to 97.00)	100.80 (IQR: 94.00 to 108.00)	112.30 (IQR: 103.50 to 122.40)	**< 0.01***
Body Mass Index (kg/m2)	24.40 (IQR: 22.10 to 27.02)	28.67 (IQR: 26.30 to 31.40)	34.06 (IQR: 30.50 to 38.70)	**< 0.01***
Weight (kg)	67.70 (IQR: 59.20 to 77.50)	81.10 (IQR: 71.50 to 91.20)	95.40 (IQR: 82.10 to 110.30)	**< 0.01***
Diabetes	8.95 (95%CI: 8.44 to 9.48)	20.88 (95%CI: 20.16 to 21.62)	38.97 (95%CI: 37.96 to 39.99)	**< 0.01***

Hypertension	49.66 (95%CI: 48.72 to 50.60)	66.47 (95%CI: 65.61 to 67.31)	76.40 (95%CI: 75.51 to 77.28)	**< 0.01***
Ethnicity				**< 0.01***
Mexican American	12.99 (95%CI: 12.41 to 13.60)	17.88 (95%CI: 17.23 to 18.56)	17.60 (95%CI: 16.85 to 18.38)	
Hispanic	8.12 (95%CI: 7.65 to 8.62)	8.93 (95%CI: 8.45 to 9.44)	7.49 (95%CI: 6.98 to 8.04)	
Caucasian	48.01 (95%CI: 47.13 to 48.90)	44.23 (95%CI: 43.38 to 45.10)	43.57 (95%CI: 42.57 to 44.57)	
African American	19.84 (95%CI: 19.14 to 20.55)	19.83 (95%CI: 19.15 to 20.53)	24.92 (95%CI: 24.06 to 25.80)	
Other Race	11.04 (95%CI: 10.50 to 11.61)	9.12 (95%CI: 8.64 to 9.64)	6.41 (95%CI: 5.94 to 6.93)	
Annual Household Income				**< 0.01***
<US$10,000	7.65 (95%CI: 7.14 to 8.19)	6.71 (95%CI: 6.25 to 7.20)	7.64 (95%CI: 7.08 to 8.24)	
US$10,000 – 24,999	25.35 (95%CI: 24.50 to 26.21)	25.24 (95%CI: 24.42 to 26.07)	27.17 (95%CI: 26.21 to 28.15)	
US$25,000 – 44,999	23.74 (95%CI: 22.92 to 24.59)	24.98 (95%CI: 24.17 to 25.82)	25.53 (95%CI: 24.59 to 26.49)	
US$45,000 – 74,999	22.14 (95%CI: 21.34 to 22.97)	22.22 (95%CI: 21.44 to 23.02)	20.74 (95%CI: 19.87 to 21.64)	
≥US$75,000	21.12 (95%CI: 20.33 to 21.93)	20.85 (95%CI: 20.09 to 21.63)	18.92 (95%CI: 18.08 to 19.78)	

Legend: IQR, Interquartile Range; 95%CI, 95% Confidence Interval; AST, Aspartate Aminotransferase; ALT, Alanine Aminotransferase; GGT, Gamma-Glutamyl Transferase; LDL, Low Density Lipoprotein; HDL, High Density Lipoprotein * bolded p-value ≤0.05 denotes statistical significance
